# The human gut bacteria *Christensenellaceae* are widespread, heritable, and associated with health

**DOI:** 10.1186/s12915-019-0699-4

**Published:** 2019-10-28

**Authors:** Jillian L. Waters, Ruth E. Ley

**Affiliations:** Department of Microbiome Science, Max Planck Institute for Developmental Biology, Max-Planck-Ring 5, 72076 Tuebingen, Germany

## Abstract

The *Christensenellaceae*, a recently described family in the phylum *Firmicutes*, is emerging as an important player in human health. The relative abundance of *Christensenellaceae* in the human gut is inversely related to host body mass index (BMI) in different populations and multiple studies, making its relationship with BMI the most robust and reproducible link between the microbial ecology of the human gut and metabolic disease reported to date. The family is also related to a healthy status in a number of other different disease contexts, including obesity and inflammatory bowel disease. In addition, *Christensenellaceae* is highly heritable across multiple populations, although specific human genes underlying its heritability have so far been elusive. Further research into the microbial ecology and metabolism of these bacteria should reveal mechanistic underpinnings of their host-health associations and enable their development as therapeutics.

## Introduction

The composition of the human gut microbiome is now well established as a factor important to human health conditions, including metabolic, pathogen, and immune-related diseases [1]. Its composition varies substantially between individuals and populations due to local, personal, and stochastic factors. The high inter-individual variability of the gut microbiome has challenged efforts to define what constitutes a healthy versus an unhealthy microbiome. Indeed, community composition alone is generally not a good predictor of disease state [2]. The contribution of specific taxa, their metabolic pathways, and their interactions to human health is a new priority for microbiome research [3], and this deeper understanding of the microbiome will be necessary for the development of evidence-based microbial therapeutics [4–6]. Given that thousands of microbial species and strains live in the gut, one challenge is to identify targets for further investigation and development.

**Fig. 1:**
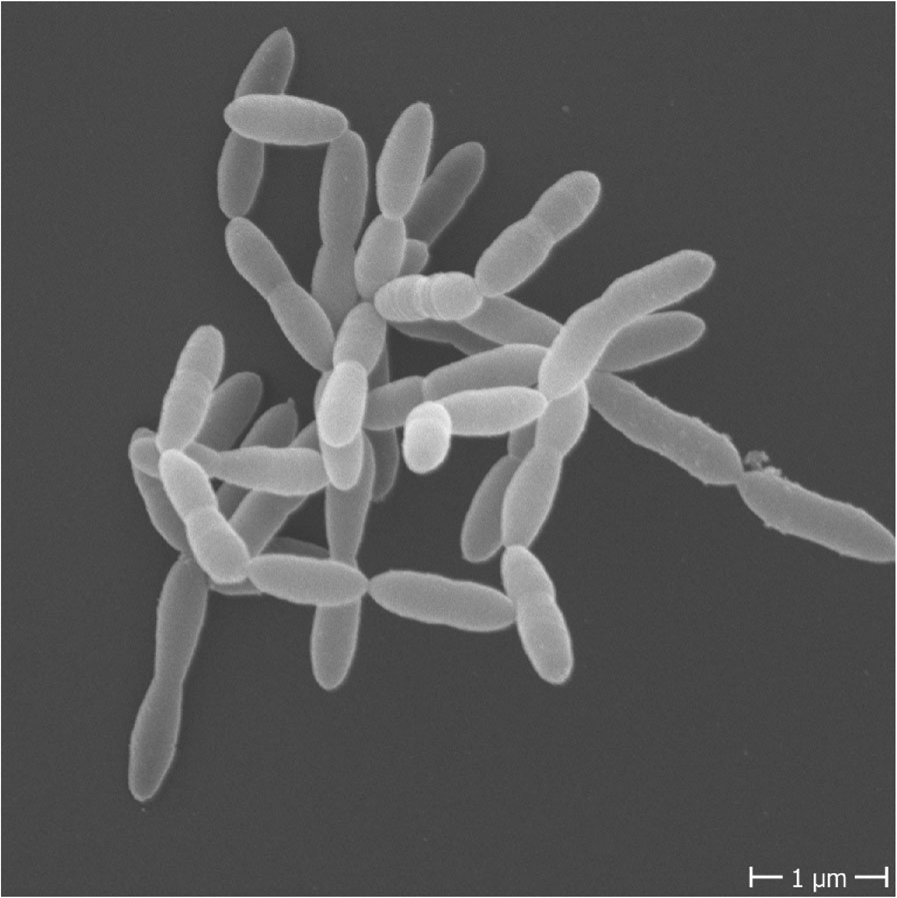
Cell morphology of *Christensenella minuta. C. minuta* (DSM22607) was grown in supplemented brain heart infusion to reach full turbidity, approximately 72 h. Cells were washed twice and subsequently resuspended in phosphate buffered saline prior to submission to the electron microscopy facility at the Max Planck Institute for Developmental Biology

Here, we focus on the family *Christensenellaceae*, within the *Firmicutes* phylum of *Bacteria*, due to its emergence as a health-related group. First encountered from 16S rRNA gene sequences alone, the family was named in 2012 after an isolate named *Christensenella minuta* (pictured in Fig. 1), cultivated from the feces of a healthy Japanese male [7]. Members of this family of *Firmicutes* are, with a few exceptions, increasingly revealing themselves as associated with a healthy phenotype in humans. Because of the relatively recent naming and phylogenetic placement of the *Christensenellaceae* family (Box 1), it was not discussed in the literature prior to a few years ago. And since representatives of this family were only recently isolated (Box 2), little is known about its ecology outside of what can be inferred from its associations with host factors and other microbiota (Box 3). Here, we review the literature to date, focusing on consistent trends that associate *Christensenellaceae* with parameters of human health. Taken together, these various observations strongly argue for further investigation into the *Christensenellaceae.*

**Box 1:** Discovery and phylogenetic classification of the *Christensenellaceae*

**Fig. 2:**
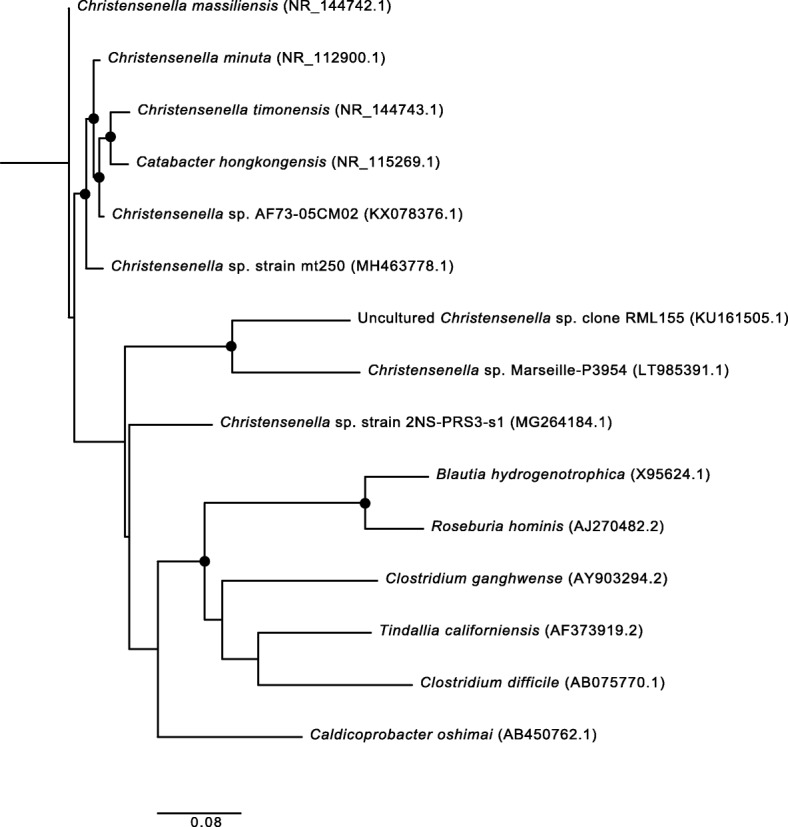
Phylogenetic relatedness of *Christensenellaceae*. Full length 16S rRNA gene sequences were obtained from NCBI and aligned using MAFFT. Accession numbers for each sequence are provided in parentheses. Bootstrap values (> 50%) are expressed as a percentage for 100 iterations. A maximum likelihood tree was built using RaxML with a general time reversible evolutionary model, and *B. thetaiotaomicron* was selected as the outgroup for rooting the tree. The scale bar represents substitutions per site

The family *Christensenellaceae* belongs to the bacterial phylum *Firmicutes*, the phylogenetically diverse and predominant phylum of the human gut microbiome. The name *Christensenellaceae* is derived from the isolate named *Christensenella minuta* (pictured in Fig. 1), which was first cultivated from the feces of a healthy Japanese male by Morotomi and colleagues and published in 2012 [7]. This isolate was named to honor the Danish microbiologist Henrik Christensen, and the species designated “minuta”, due to the small size of the cell (0.8–1.9 μM) and the colonies it forms on agar plates (only 0.1 mm in diameter). In their species description, Morotomi et al. compared *C. minuta*’s full length 16S rRNA against publicly available databases and identified *Caldicoprobacter oshimai*, a bacterium in the family *Caldicoprobactereaceae* (*Clostridiales*), as the closest relative, with 86.9% pairwise ID. Other related taxa included *Tindallia californiensis* (86.3% ID) and *Clostridium ganghwense* (86.1% ID), both of which are in the family *Clostridiaceae* in the phylum *Firmicutes*. They did note that other sequences were identified with matches greater than 98% ID; however, these were unclassified taxa from other 16S rRNA gene diversity surveys. *C. minuta* was designated to represent a novel family, *Christensenellaceae*, in the order *Clostridiales* in the phylum *Firmicutes* [7]. A closely related bacterium, *Catabacter hongkongensis,* was described in 2007 [8]*.* The 16S rRNA genes of *C. minuta* and *Catabacter hongkongensis* share 96.5% sequence identity, suggesting the two should be in the same family, and possibly the same genus [9] (Fig. 2). As a result, some databases use the family name *Catabacteriaceae*, some use *Christensenellaceae*, and some studies include both as two distinct families. The family name *Christensenellaceae*, however, is now considered with standing in nomenclature [10]. The Genome Taxonomy Database, a recent taxonomy developed by Phil Hugenholtz and colleagues that is based on whole genome comparisons rather than 16S rRNA gene sequences for reconstructing phylogeny, supports that *Christensenella* and *Catabacter* are separate genera in the family *Christensenellaceae*, within a new order *Christensenellales* [11].

**Box 2:** Cultured isolates of the family *Christensenellaceae* (2019)

The first isolate, *Christensenella minuta* (DSM 22607)*,* was isolated from the feces of a healthy Japanese male. It is strictly anaerobic, non-sporulating, non-motile, and described as Gram-negative [7]. Intriguingly, others have described it as Gram-positive [12], which is also consistent with our unpublished observations. A Gram-positive cell wall is consistent with its classification as belonging to the phylum *Firmicutes*, which includes predominantly Gram-positive bacteria. However, *C. minuta* is able to produce small amounts of lipopolysaccharide, an attribute that is more typical of, but not exclusive to, Gram-negative bacteria [13]. Morotomi and colleagues demonstrated that *C. minuta* produces the short chain fatty acids acetate and butyrate, and is saccharolytic, with the ability to utilize arabinose, glucose, mannose, rhamnose, salicin, and xylose. *C. minuta* was negative for many of the standard biochemical assays used for characterization, which included catalase, oxidase, esculin and gelatin hydrolysis, indole production, and nitrate reduction [7]. The genome was published in 2017 [14], and is estimated as 2.94 Mb with 51.5% G + C content. *Catabacter hongkongensis* (DSM 18959)*,* first described in 2007, was isolated from the blood of patients who developed bacteremia in Canada and Hong Kong. *Catabacter hongkongensis* is described as strictly anaerobic, non-sporulating, and Gram-positive [8]. In contrast to the other *Christensenella* isolates, *Catabacter hongkongensis* is in fact motile. *Catabacter* has been associated with bacteremia in at least 12 additional instances, and there may be more due to the difficulty in many chemical-based methods of accurately identifying *Catabacter hongkongensis* [15–17]. *Catabacter hongkongensis* has a similar saccharolytic profile to *C. minuta*, with the exception of glycerol and rhamnose utilization depending on the isolate, and it was not able to utilize salicin. *Catabacter hongkongensis* differs from *C. minuta* in that it is catalase positive. Like *C. minuta,* it was negative for oxidase, esculin and gelatin hydrolysis, indole production, and nitrate reduction [8]. No short chain fatty acid production has been reported for *Catabacter.* The genome for this bacterium was published in 2015, and is 3.2 Mb with 48.5% G + C content. Annotation of the genome supported that *Catabacter hongkongensis* is motile, and the authors identified a number of antibiotic resistance genes, which may contribute to its pathogenicity [18]. *Christensenella massiliensis* (DSM 102344) and *Christensenella timonensis* (DSM 102800)*,* both isolated from the feces of a diabetic patient in Marseilles, France, are described as strictly anaerobic, non-motile, non-sporulating, and Gram-negative, similar to *C. minuta* [19, 20]. Although 16S rRNA gene sequence comparisons place *C. timonensis* within the *Christensenella* genus (> 97% identity to *C. minuta*), whole genome taxonomy indicates it belongs to a genus distinct from both *Christensenella* and *Catabacter* [11]. No characterization of these isolates has been reported.

**Box 3:** Ecological role of the *Christensenellaceae* in the human gut

Based on Morotomi’s observations, *C. minuta* ferments glucose to acetate and butyrate under anaerobic conditions [7], which indicates it ferments sugars in the gut to short chain fatty acids and other fermentation products such as H_2_ and CO_2_. Goodrich et al. reported that the *Christensenellaceae* form the hub of a co-occurrence network with other microbiota, including methanogens (archaea of the family *Methanobacteriaceae*) [21]. Co-occurrence of *Christensenellaceae* and *Methanobacteriaceae* across individuals has been reported elsewhere [22, 23]. The *Methanobacteriaceae* include *Methanobrevibacter smithii*, the predominant methanogen in the human gut. Given that *M. smithii* uses fermentation products (e.g., H_2_ and CO_2_) to produce methane, the co-occurrence with *Christensenellaceae* may represent a H_2_-based syntrophy.	

## *Christensenellaceae* is ubiquitous among humans and other animals

Most of what is known about the family *Christensenellaceae* comes from 16S rRNA gene surveys of the microbiome obtained from feces of humans and other animals. Given that *Christensenellaceae* 16S rRNA gene sequences were relatively recently included in reference databases, only microbiome studies published since 2013 report this taxon. Two cultured isolates, *Christensenella minuta* and *Catabacter hongkongensis*, have published genomes [14, 18], and genomes constructed during metagenomic assemblies are increasingly available. At the time of writing this review, there are 11 *Christensenellaceae* genomes in the Genome Taxonomy Database and 89 genomes for the order *Christensenellales* (Box 1) [11]. A phylogeny of 9 members of the Christensenellaceae, based on full length 16S rRNA gene sequences available in NCBI, is shown in Fig. 2. Surveying the post-2013 literature, it is evident that members of the *Christensenellaceae* are cosmopolitan inhabitants of the animal gut (Table 1), with a likely preference for the distal colon [44], which is consistent with its fermentative activities (detailed in Box 3) [7].

**Table 1:** Christensenellaceae has a wide range of hosts in the animal kingdom

Phylum	Class	Order	Family	Genus	Species	Common name	Reference(s)
Chordata	Aves	Casuariiformes	Casuariidae	*Dromaius*	*novaehollandiae*	Emu	[24]
		Galliformes	Phasianidae	*Gallus*	*gallus*	Chicken	[25]
				*Coturnix*	*japonica*	Japanese quail	[26]
		Struthioniformes	Struthionidae	*Struthio*	*camelus*	Ostrich	[27]
	Mammalia	Artiodactyla	Bovidae	*Bos*	*frontalis*	Gayal	[28]
					*taurus*	Cow	[28, 29]
				*Capra*	*aegagrus hircus*	Goat	[30]
				*Syncerus*	*caffer*	African Buffalo	[28]
				*Ovis*	*aries*	Sheep	[31]
			Camelidae	*Camelus*	*bactrianus*	Bactrian camel	[32]
					*dromedarius*	Dromedary camel	[33]
			Cervidae	*Cervus*	*nippon*	Sika Deer	[34]
					*elaphus*	Red deer	[28]
			Giraffidae	*Giraffa*	*camelopardalis*	Giraffe	[28]
			Suidae	*Sus*	*scrofa*	Pig	[35, 36]
		Carnivora	Canidae	*Canis*	*lupus*	Dog	[37]
			Felidae	*Felis*	*catus*	Cat	[38]
		Diprotodontia	Vombatidae	*Lasiorhinus*	*latifrons*	Southern hairy-nosed wombat	[39]
		Lagomorpha	Leporidae	*Oryctolagus*	*cuniculus*	Rex rabbit	[40]
		Perissodactyla	Equida	*Equus*	*caballus*	Horse	[28, 41]
				*Equus*	*quagga*	Zebra	[28]
		Primates	Cercopithecidae	*Cercopithecus*	*ascanius* ^*a*^	Red-tailed monkey	[42]
					*wolfi* ^*a*^	Wolf’s mona monkey	[42]
					*neglectus* ^*a*^	De Brazza’s monkey	[42]
				*Macaca*	*mulatta*	Rhesus Macaque	[43]
				*papio*	*anubis*	Baboon	[44]
		Rodentia	Cricetidae	*Cricetus*	*cricetus*	European hamster	[28]
				*Microtus*	*californicus scirpensis*	Amargosa vole	[45]
			Muridae	*Mus*	*musculus*	Mouse	[46]
				*Rattus*	*norvegicus*	Rat	[47]
		Sirenia	Dugongidae	*Dugong*	*dugon*	Dugong	[48]
			Trichechidae	*Trichechus*	*Manatus manatus*	Antillean manatee	[49]
	Reptilia	Squamata	Lacertidae	*Podarcis*	*lilfordi*	Lilford’s wall lizard	[50]
			Liolaemidae	*Liolaemus*	*parvus*	Lesser smooth-throated lizard	[51]
					*ruibali*	Ruibal’s tree iguana	[51]
		Testudines	Testudinidae	*Gopherus*	*polyphemus*	Gopher tortoise	[52]
Anthropoda	Insecta	Coleoptera	Scarabaeidae	*Holotrichia*	*parallela*	Large black chafer	[53]
		Blattodea	Blaberidae	*Diploptera*	*punctata*	Pacific beetle cockroach	[54]
				*Pycnoscelus*	*surinamensis*	Surinam cockroach	[55]

^a^*Christensenellaceae* is listed as detected in the *Cercopithecus* genus, without further species detail. The three species listed were studied in McKenzie et al. [42]

In humans, the family comprises on average 0.01% of the fecal microbiota [21]. Its fine-scale distribution along the human gastrointestinal tract remains to be clarified; but in addition to feces, *Christensenellaceae* has been detected in human colonic mucosa, ileum, and appendix, and there is also suggestive evidence of airway colonization [21, 56–59]. The family *Christensenellaceae* is widespread across human populations, and is reported from subjects inhabiting North America [60–62], South America [63, 64], Europe [21, 65], Asia [66, 67], Africa [68–70], and Australia [71].

Within human populations, traits associated with different relative abundances of *Christensenellaceae* include ethnicity and sex. For instance, a recent study of > 2000 individuals with various ethnicities residing in Amsterdam, Deschasaux et al. reported that Dutch subjects harbored the greatest relative abundances of *Christensenellaceae* [72]. Similarly, Brooks et al. compared microbiome variation between ethnicities in 1673 people residing in the USA and reported that *Christensenellaceae* was overall less represented in fecal samples of Asian-Pacific Islanders relative to other ethnicities [60]. A greater relative abundance of *Christensenellaceae* in women compared to men was also observed [60], and similar observations have been reported in animals [26, 73, 74]. The underlying causes of these ethnic and sex differences are unclear.

*Christensenellaceae* has been associated with human longevity, based on the observation that the relative abundance of *Christensenellaceae* is greater in centenarians and supercentenarians in comparison to younger individuals in populations in China [75, 76], Italy [77], and Korea [78]. Positive associations of *Christensenellaceae* with age have also emerged from studies with relatively young individuals across multiple geographic locations [60, 68, 79–82] (Table 2). Given that none of these studies followed the same individuals over time, the association with age could reflect a cohort effect rather than an age effect. For example, dietary patterns that vary by age may influence this association (see below), or individuals born earlier may have always harbored greater levels of *Christensenellaceae* compared to those born later.

**Table 2:** The relative abundance of *Christensenellaceae* increases with age

Country	Sample size of cohort	Age	Sex	Reference
		(mean ± std. dev.) *^, #^	(% male/% female)	
China	168	93.3 (90-102) Long-living people^#^	37/63	[75]
		61.6 (24-83) Young^#^	52/48	
China	24	104 (100-108) Centenarians*	38/62	[76]
		92 (85-89) Bama elderly*	38/62	
		83 (80-92) Nanning elderly*	50/50	
Italy	69	106.2 (105-109) Semi-supercentenarians^#^	25/75	[77]
		100.4 (99-104) Centenarians^#^	7/93	
		72.5 (65-75) Elderly^#^	47/53	
		30.5 (22-48) Adults^#^	47/53	
Korea	47	98.9 ± 3.4 Centenarians	33,147	[78]
		73.6 ± 3.6 Elderly	59/41	
		34.3 ± 6.5 Adults	67/33	
Korea	57	25-65 (no other participant info or table)	54/46	[82]
USA	1673	40.2 ± 9.7^a^	52/48^a^	[60]
USA	28	49.5 (20-82)*	54/46	[79]
Nigeria	30^b^	Infant-85^c^	NA	[68]
United Kingdom	2764^d^	59.5 ± 12.3	32,813	[81]
Canada	41	24.3 ± 3.7^e^	54/46	[80]

* In these studies age is reported as median (age range)

^#^ In these studies age is reported as average (age range)

^a^ Metadata were only reported for the American Gut Participants (*n* = 1375) as participant data for the Human Microbiome Project is restricted access

^b^ These findings only pertain to the urban dwelling Nigerians from this study

^c^ A median or average for age groups was not provided. Infants were defined as < 3 years of age (*n* = 12) and adults were 5-85 (*n* = 18)

^d^ These values only pertain to the analysis in the TwinsUK cohort in this paper

^e^ These values are reported for the *AVG* cohort with regard to cardiorespiratory fitness, but is reflective of all study participants. Total age range for all participants is between 18 and 35 years

## The *Christensenellaceae* are linked to host genetic variation

Host genotype is estimated to influence 30–60% of the variation in the relative abundance of *Christensenellaceae* across individuals [21, 62, 66, 83]. Of the hundreds of taxa in the gut, the family *Christensenellaceae* is consistently identified as among the most highly heritable. This means that a significant proportion of the variance in the relative abundance of the family across a population can be attributed to genetic factors. Heritability refers to the genetic predisposition of a quantitative trait: for example, height is heritable, because this trait is largely genetically determined. Heritability calculations take into account quantitative measures of the trait (such as relative abundance) and should not be confused with whether the *Christensenellaceae* are inherited (i.e., vertically transmitted) from family members, which is not known.

Goodrich et al. first identified the *Christensenellaceae* as heritable in a well-powered (*n* = 977) study of monozygotic and dizygotic twins from the UK [21]. A remarkable 40% of the variation between individuals in the relative abundance of the family *Christensenellaceae* could be attributed to host genetic factors. A more fine-grained analysis of species-level operational taxonomic units (OTUs) showed that just a few *Christensenellaceae* OTUs were driving the heritability of the family [21]. Other studies of heritability employing the same population have observed similar results, whether the analysis was specific to species-level OTUs or when analyzing modules of co-occurring microbes [81, 84]. So far, the *Christensenellaceae* have not been included in the analysis of heritability based on shotgun metagenome data, due to the absence of genomes for this family in the reference databases used [85].

The high heritability of the *Christensenellaceae* has been corroborated in other human populations. Goodrich et al. had confirmed its heritability in two previous studies involving twin pairs from the USA [21, 61, 86]. Additionally, Lim et al. evaluated microbiome heritability in a Korean cohort of 655 individuals and identified *Christensenellaceae* as heritable. In a Canadian cohort (*n* = 270), it was again identified as among the most highly heritable taxa [62]. Together, these observations across multiple populations indicate that the heritability of the *Christensenellaceae* is a widely shared trait. That individuals are genetically predisposed to harbor a high or low relative abundance of these bacteria may be a generalizable human trait.

So far, attempts to identify the genetic factors that account for the high heritability of *Christensenellaceae* by genome-wide association (GWA) have not succeeded [83]. These studies are generally underpowered, given the millions of tests conducted simultaneously (i.e., testing all genetic variants against all microbiome traits), and the necessity to correct for false positives [87]. An alternative to GWA is to take a candidate gene approach, restricting the analysis to genes with interesting functions. For instance, Zakrzewski et al. examined the relationship between a SNP in the interleukin 23 receptor (ILR23) gene and the microbiome of mucosal biopsies from the ileum and rectum. The A allele of this variant has been associated with a reduced risk of ileal Crohn’s disease (CD). Within a population of individuals with no signs of CD or other gastrointestinal disorders, a significantly greater relative abundance of *Christensenellaceae* was detected in the feces of individuals harboring the protective allele (AG genotype) compared to the population with the GG genotype [56]. How the IL23R genotype may affect members of the gut microbiota remains to be clarified.

*Christensenellaceae* has also been associated with the fucosyltransferase 2 (*FUT2*) gene, which encodes an enzyme responsible for ABO blood group antigens that are expressed on the intestinal surface as well as secreted. Non-secretors (AA genotype) have an elevated risk for CD, while secretors (AG or GG) are less likely to develop CD [88]. A re-analysis of healthy individuals studied in [88] showed that secretors harbored relatively more of this family compared to non-secretors (*n* = 24) [21]. It is important to note that in this case a targeted approach was used, and subsequent studies associating the microbiome with *FUT2* do not reach this same conclusion. When Davenport et al. also did this analysis in UK twins (*n* = 1503), where heritability of *Christensenellaceae* was first reported, no link between *Christensenellaceae* and secretor status was found [89], which is consistent with the results of Turpin et al. in a cohort of 1190 healthy individuals [90].

The *Christensenellaceae* may interact with host genetic status to affect risk of colorectal cancer (CRC). Le Gall et al. reported elevated *Christensenellaceae* in healthy controls relative to individuals with CRC (*n* = 50 age- and sex-matched individuals per group) [91], yet Yazici et al. observed that the relative abundance of *Christensenellaceae* in stool was higher on average in African-American CRC patients compared to controls [92]. Furthermore, using tumor and healthy mucosal tissue biopsies from 44 patients with five different loss-of-function mutations in CRC, Burns et al. observed that the association of *Christensenellaceae* with CRC was dependent on the type of mutation present [58]. These findings may offer an explanation for the inconsistent patterns of *Christensenellaceae* abundance with respect to CRC status. However, whether the *Christensenellaceae* participate in CRC pathology remains to be ascertained. While associations between *Christensenellaceae* and host genotypes remain to be reproduced, they suggest that health/disease promotion by these genotypes may be mediated in part through promotion of the *Christensenellaceae*.

## The *Christensenellaceae* are linked to metabolic health

### Body composition and metabolic health

Body mass index (BMI) was the first host phenotype associated with the relative abundance of *Christensenellaceae* in the gut. Goodrich et al. observed that *Christensenellaceae* was significantly enriched in individuals with a normal BMI (18.5–24.9) compared to obese individuals (BMI ≥ 30) [21]. Since this initial observation, the association of *Christensenellaceae* with a normal BMI has been corroborated repeatedly in populations from a number of countries that included adult men and women of various ages (Table 3). Consistent with its association with leanness, *Christensenellaceae* have been shown to increase after diet-induced weight loss [100]. Although obese and lean subjects can often be differentiated using aspects of microbial ecology of the gut, these aspects (e.g., alpha-diversity, or abundances of phyla) have differed between studies [101]: the link between *Christensenellaceae* and BMI therefore stands as the strongest corroborated association between the gut microbiome and BMI.

**Table 3:** Global associations of *Christensenellaceae* with a healthy body mass index

Country	Sample size of cohort	Age (mean ± std. dev.)*	Sex (% male/% female)	Reference
USA	154	15 (21-32)*^,a^	0/100	[61]
USA	599	62.7 ± 7.7^b^	54/46	[93]
USA	1673	40.2 ± 9.7^c^	52/48 ^c^	[60]
Mexico	138	9.9 ± 1.72^b^	58/42	[94]
United Kingdom	977	60.6 ± 0.3	2/98	[21]
United Kingdom	2764^d^	59.5 ± 12.3	11/89	[81]
Spain	39	14.8 (13-16)*	49/51	[95]
Netherlands	893	44.7 ± 12.9	43/57	[96]
Norway	384	48 (23-82)*	42/58	[97]
Norway	169	30 (27-34)*	0/100	[98]
Korea	655	47.0 ± 12.2	42/58	[66]
Korea	1274	45.7 ± 9.0	64/36	[99]
Japan	516	52.4 ± 13.4	37/63	[67]

* In these studies age is reported as median (range)

^a^ 49 participants are mothers of the twins, for which no age is reported

^b^ These values are reported for the healthy weight cohort, but is reflective of all study participants

^c^ Metadata were only reported for the American Gut Participants (*n* = 1375) as participant data for the Human Microbiome Project is restricted access

^d^ These values only pertain to the analysis in the TwinsUK cohort in this paper. Other studies were included, but *Christensenellaceae* was not reported

BMI is a proxy for adiposity, and consistent with reports linking levels of *Christensenellaceae* with BMI, studies in which adiposity is more directly measured have also noted strong associations with the abundance of *Christensenellaceae* in the gut. For instance, Beaumont et al. correlated adiposity measures, determined using dual x-ray absorptiometry (DEXA), with the microbiome in a study of 1313 UK twins. At the family level, the most significant association was with *Christensenellaceae*, which negatively correlated with visceral fat mass [84], a type of fat that is considered a cardiometabolic risk factor. A similar observation was made by Hibberd et al., who reported significant negative correlations of *Christensenellaceae* with trunk fat and android fat [102]. Additionally, *Christensenellaceae* has been negatively correlated with waist circumference and waist to hip ratio, which are direct markers of central adiposity [66, 102–104].

In addition to its association with body fat measures, *Christensenellaceae* is negatively correlated with serum lipids in several studies. In the Dutch LifeLines DEEP cohort (*n* = 893), Fu et al. reported a negative correlation of *Christensenellaceae* with BMI, together with a strong association with low triglyceride levels and elevated levels of high density lipoprotein (HDL, or “good cholesterol”) [96]. Other groups have also reported that *Christensenellaceae* is associated with reduced serum triglycerides [66, 102, 104]. Similarly, this family is also negatively associated with total cholesterol, low density lipoprotein (LDL; or “bad cholesterol”), and apolipoprotein B, a component of LDL particles [94, 102].

*Christensenellaceae* is reported as depleted in individuals with metabolic syndrome (MetS) compared to healthy controls [66, 104]. In addition to excess visceral fat, MetS includes other risk factors such as dyslipidemia and impaired glucose metabolism, and is a risk factor for type 2 diabetes and cardiovascular disease. *Christensenellaceae* was identified in a cohort of 441 Colombians as positively associated with a lower cardiometabolic risk score [103], and others report it is negatively correlated with blood pressure [66, 104, 105], which is often elevated in MetS [106]. *Christensenellaceae* has also been associated with healthy glucose metabolism [66, 107] and *Christensenellaceae* OTUs are reduced in individuals with pre-type 2 diabetes [65]. Given that a high BMI, impaired glucose metabolism, dyslipidemia, and other aspects of MetS are comorbidities, it is not surprising that *Christensenellaceae* inversely tracks with many of these conditions. The mechanism underlying its negative association with MetS remains to be elucidated.

Metabolic disorders are often linked to dietary patterns. The *Christensenellaceae* appear to be responsive to diet, and evidence points to a role in protein and fiber fermentation. On a coarse level, large-scale diet studies have associated *Christensenellaceae* with healthy dietary habits low in refined sugar and high in consumption of fruit and vegetables [108–110]. *Christensenellaceae* is reported higher in relative abundance in humans with an omnivorous diet, relative to vegetarians [71, 111], and has also been associated with dairy consumption [112]. In a more direct link, *Christensenellaceae* has been shown to respond rapidly to an increase in animal products in the diet [113]. Furthermore, *Christensenellaceae* has been positively associated with gut metabolites typical of protein catabolism and dietary animal protein [114–116]. *Christensenellaceae* has also been reported to increase in human dietary interventions involving prebiotic fibers such as resistant starch 4, galacto-oligosaccharide, and polydextrose [22, 102, 112]. Similar observations have also been made in rodent models [117–119]. Taken together, these studies indicate that the association of *Christensenellaceae* with health parameters may in part be due to its association with a diet high in protein and fiber.

To test for a causal role for *Christensenellaceae* in metabolic disease while controlling for diet, Goodrich et al. selected an obese human donor based on almost undetectable levels of *Christensenellaceae* in the microbiome, and performed fecal transfers to germfree mice that were fed the same fiber-rich chow, but otherwise only differed by whether or not the obese human microbiome inoculum was amended with *C. minuta*. These experiments showed that amendment with *C. minuta* reduced the adiposity gains of mice compared to those that received unamended stool (or stool amended with heat-killed *C. minuta*) [21]. The mechanism underlying the protective effect of *C. minuta* against excess adiposity gain remains to be elucidated, but may involve re-modeling the microbial community, as a shift in diversity was observed when *C. minuta* was added. These experiments demonstrated that the activity of *C. minuta* in the gut microbiome can affect host body composition even when diet is controlled for, possibly via interactions with other members of the microbiota. Indeed, the ecological role of members of the *Christensenellaceae* and their function in the gut in general remains to be better understood (Box 3).

### Inflammation and transit time

In a meta-analysis of inflammatory bowel disease (IBD) that included over 3000 individuals, Mancabelli et al. reported *Christensenellaceae* as one of five taxa considered a signature of a healthy gut [120]. Indeed, *Christensenellaceae* were consistently depleted in individuals with Crohn’s disease [121–129] and ulcerative colitis [97, 122, 125, 129, 130], the two major sub-types of IBD. In irritable bowel syndrome (IBS), a gastrointestinal disorder characterized by abdominal pain and abnormal bowel movements, a higher relative abundance of *Christensenellaceae* in healthy controls relative to individuals with IBS has been reported in several studies [131–134]. Several studies have also noted a positive correlation of *Christensenellaceae* and longer transit time or even constipation [67, 114, 133, 135, 136]. Thus, the *Christensenellaceae* appear to be depleted in conditions associated with inflammation and fast transit time.

Given *Christensenellaceae*’s link with transit time, it is perhaps not surprising that the family has been linked to affective disorders that impact gut motility. For instance, gastric dysfunction, particularly constipation, affects approximately two-thirds of patients with Parkinson’s disease (PD) and multiple sclerosis (MS) [137, 138]. Studies have noted a greater relative abundance of *Christensenellaceae* in PD and MS patients relative to healthy controls [139–142]. Since diet is also related to gut transit time, the effects of diet, host status, and host genetics remain to be carefully disentangled to better understand how levels of the *Christensenellaceae* are controlled.

## Prospectus

The family *Christensenellaceae* is a relatively recently described bacterial family that is highly heritable and shows compelling associations with host health. Its strong ties to host health have warranted the suggestion that cultured representatives of the *Christensenellaceae,* such as *C. minuta*, should be considered for use as a therapeutic probiotic for the improvement of human health [143]. However, the functional role of *Christensenellaceae* in the gut remains to be understood. While the collection of associations between *Christensenellaceae* and host health parameters continues to grow, allowing inferences about the role of these bacteria, they remain to be studied experimentally. Genomes offer a powerful platform for generating hypotheses regarding the metabolic capacity of the *Christensenellaceae*, but further functional characterization in vitro and in vivo will be necessary to fully characterize the role of *Christensenellaceae* in the gut. The ecological role of members of the *Christensenellaceae*, their interactions with other members of the microbiome and with the host and host diet, all remain to be better understood if these intriguing microbes are to be harnessed fully to improve human health.

## Data Availability

16S rRNA gene sequences used to construct the phylogenetic tree were obtained from NCBI (https://www.ncbi.nlm.nih.gov/). Accession numbers for each sequence are in parentheses in Fig. 2.
